# Prevalence of Sarcopenic Obesity Using Different Definitions and the Relationship With Strength and Physical Performance in the Canadian Longitudinal Study of Aging

**DOI:** 10.3389/fphys.2020.583825

**Published:** 2021-01-21

**Authors:** Sarah A. Purcell, Michelle Mackenzie, Thiago G. Barbosa-Silva, Isabelle J. Dionne, Sunita Ghosh, Mario Siervo, Ming Ye, Carla M. Prado

**Affiliations:** ^1^Human Nutrition Research Unit, Department of Agricultural, Food, and Nutritional Science, University of Alberta, Edmonton, AB, Canada; ^2^Division of Endocrinology, Metabolism, and Diabetes, School of Medicine, University of Colorado, Aurora, Colorado, CO, United States; ^3^Section of Nutrition, Department of Pediatrics, School of Medicine, University of Colorado, Aurora, Colorado, CO, United States; ^4^Department of General Surgery, Faculty of Medicine, Federal University of Pelotas, Pelotas, Brazil; ^5^Department of Obstetrics and Gynecology, Catholic University of Pelotas, Pelotas, Brazil; ^6^Department of Kinanthropology, Faculty of Physical Activity Sciences, Université de Sherbrooke, Sherbrooke, QC, Canada; ^7^Research Centre on Aging, Sherbrooke, QC, Canada; ^8^Department of Oncology, Faculty of Medicine and Dentistry, University of Alberta, Edmonton, AB, Canada; ^9^School of Life Sciences, The University of Nottingham Medical School, Queen’s Medical Centre, Nottingham, United Kingdom; ^10^School of Public Health, University of Alberta, Edmonton, AB, Canada

**Keywords:** Geriatrics, community-dwelling older individuals, muscle strength, older people, physical function, body composition, CLSA

## Abstract

Sarcopenic obesity is associated with several negative health outcomes. However, the prevalence of this condition – and the relationship to physical performance parameters – varies across definitions. The aim of this cross-sectional investigation was to describe the prevalence of sarcopenic obesity using different published definitions and their relationship with handgrip strength and walking speed in older Canadian adults. Individuals aged 65+ in the Canadian Longitudinal Study on Aging (*n* = 11,803; 49.6% male, 50.4% female) were included. Body composition was measured using dual X-ray absorptiometry. Sarcopenic obesity was defined using 29 definitions. Low handgrip strength was identified as < 27 kg in males and < 16 kg in females and poor physical performance was defined as gait speed ≤ 0.8 m/s. The prevalence of sarcopenic obesity ranged from 0.1 to 85.3% in males, and from 0 to 80.4% in females. Sarcopenic obesity was frequently associated with low handgrip strength (*p* < 0.05) in both males (14/17 definitions, 82.4%) and females (21/29 definitions, 72.4%). In very few definitions, sarcopenic obesity was associated with slow gait speed (males: 1/17 definitions [6.7%]; females: 2/29 [6.9%]). In conclusion, the prevalence of sarcopenic obesity varied greatly according to definitions and sarcopenic obesity was frequently associated with low handgrip strength.

## Introduction

Population aging and obesity are two of the greatest epidemiological trends occurring globally (Roubenoff, 2004). Currently, one in six Canadians (17.2%) is ≥ 65 years of age (Statistics Canada, 2019a); 28.0% of older Canadians have a body mass index (BMI) in the ‘obese’ category (≥ 30 kg/m^2^) and another 40.3% have a BMI in the ‘overweight’ category (≥ 25 kg/m^2^) (Statistics Canada, 2019b).

Both aging and obesity are associated with significant health problems. Morbidity of aging and obesity are both mediated in part through changes in body composition (i.e., the amounts of fat and lean tissues in the body). One such body composition alteration that occurs with senescence is decreased muscle mass. Although loss of muscle mass is an ubiquitous problem as one ages, negative consequences such as increased health care costs, physical impairment, disability, falls, fractures and frailty (Baumgartner et al., 1998; Bales and Ritchie, 2002; Janssen et al., 2004; Visser and Schaap, 2011) are only evident once a threshold of muscle loss is reached.

Low muscle mass may also occur with obesity (excess fat mass), a term called ‘sarcopenic obesity.’ This condition is a considerable public health problem with severe consequences (Roubenoff, 2004; Prado et al., 2012). The double-burden of low muscle mass and obesity may accentuate the risk of developing chronic degenerative diseases but, more importantly, it could increase the years of disability, which have a severe impact on the sustainability and efficiency of health initiatives across all levels of organizations (primary and secondary care, social support, public health and policy makers) (Roubenoff, 2004). In fact, there is evidence to suggest that individuals with sarcopenic obesity have worse morbidity, disability and mortality than either low muscle mass or obesity (Baumgartner et al., 2004; Schrager et al., 2007; Zhang et al., 2019). Moreover, obesity masks low muscle mass, making its diagnosis and identification of clinical consequences a challenge (Prado et al., 2012).

Despite the negative impact of these abnormal body composition phenotypes on the public health care system, there is no consensus on the most appropriate definition for sarcopenic obesity. Contrary to popular belief, the combination of definitions of ‘sarcopenia’ in older adults (muscle mass *and* function) with high BMI or adiposity is not widely accepted in the context of sarcopenic obesity. The lack of standardized criteria impacts awareness, resource allocation and the recognition of this condition as an important public health problem. Additionally, factors such as age, ethnicity, and geographical location all impact body composition (Lear et al., 2009; Silva et al., 2010), yet a comparison of the prevalence of sarcopenic obesity definitions has not been described in Canadians. Furthermore, which definitions of sarcopenic obesity most closely relate to strength or physical performance outcomes is unknown within this population. Therefore, the objective of the current investigation was to describe the prevalence of sarcopenic obesity using different published definitions in older Canadian adults. As an exploratory objective, we also characterized the relationship between each definition of sarcopenic obesity with handgrip strength and walking speed.

## Materials and Methods

### Study Population and Demographics

The present cross-sectional analysis used data from the Canadian Longitudinal Study on Aging (CLSA) (Raina et al., 2009; Kirkland et al., 2015). The CLSA is a large national cohort study of individuals age 45–85 that collects longitudinal data on several biological, medical, psychological, social, and economic aspects of health and disease. Individuals are randomly selected for inclusion based on age and sex strata via the Canadian Community Health Survey and provincial health care records. Individuals are included the CLSA cohort if they are between the ages of 45–85 years and able to read and speak in English or French. Those in long-term care facilities or with cognitive impairment that precludes informed consent at baseline are excluded. For the present analysis, only baseline data for males and females aged ≥ 65 years old who were part of the in-depth, nationwide cohort of the CLSA were included. Age, sex, comorbidities, and health behaviors were self-reported during an in-person interview. Participants also self-identified their ethnicity or cultural background their ancestors belonged. The following responses were considered ‘Caucasian’: Canadian, French, English, German, Scottish, Irish, Italian, Ukrainian, Dutch, Hebrew, Polish, Norwegian, Welsh, Swedish. Due to the known differences in body composition among different ethnicities (Heymsfield et al., 2016) and the small number of participants who self-identified as non-Caucasian ethnic backgrounds, only Caucasian participants were included in the present analysis.

### Anthropometrics and Body Composition

Height and weight were measured using standard procedures during a visit to the nearest data collection site. Body weight was recorded using a 140-10 Digital Physician Scale (Rice Lake Weight Systems, Rice Lake, WI, United States) after removal of shoes, headwear, and excess layers of clothing. Height was measured with a Seca 213 stadiometer (Hamburg, Germany) to the nearest 0.1 centimeter. The average of two measures of height and weight were used. Body mass index was calculated from height and weight measurements and classified according to the World Health Organization cut-points (underweight < 18.5 kg/m^2^, normal weight 18.5–24.9 kg/m^2^, overweight 25.0–29.9 kg/m^2^, and obese ≥ 30.0 kg/m^2^) (World Health Organization, 2015).

Body composition was measured using whole body dual energy X-ray absorptiometry (DXA) Hologic Discovery A^TM^ (Marlborough, MA, United States). The sum of lean soft tissue (LST) in both arms and legs was used as a measure of appendicular skeletal muscle mass (ASM; kg) and appendicular skeletal muscle index (ASMI; ASM [kg]/height [m^2^]) was calculated from this value. Although a more correct definition of this compartment would be appendicular LST as skeletal muscle is not directly measured (Heymsfield et al., 1990), we will use the common abbreviations (i.e. ASM and its derivatives) to avoid confusion with the originally published studies. Whole-body fat mass (FM) and LST including bone (fat-free mass, FFM) were also used for sarcopenic obesity assessment. Truncal fat mass (TrFM) from DXA results were also used for sarcopenic obesity definitions. Measures of body composition were expressed in absolute values (kg), adjusted for height or BMI, or as a percent of total body weight (e.g., FM [kg]/body weight [kg] x 100, percent body fat) according to selected definitions. For consistency, we referred to whole-body LST assessments as “muscle mass” assessments in this paper for simplicity.

### Sarcopenic Obesity Definitions

Definitions of ‘sarcopenia’ within aging literature (i.e., primary sarcopenia) may require the coexistence of both low muscle mass and poor physical function or performance (Cruz-Jentoft et al., 2019). However, sarcopenic obesity has rarely been defined with physical function or performance parameters and there is no consensus that including other functional indices is the best approach for identifying this condition (Donini et al., 2019). Further, the International Classification of Disease, 10th Revision (ICD-10) code for sarcopenia (M62.84) does not specify how sarcopenia should be defined. Therefore, there is no consensus for sarcopenic obesity definition. As such, sarcopenic obesity in the current investigation was defined as concurrent low muscle mass (defined by several different definitions including LST, ASM, and FFM compartments and its derivatives) and obesity. Diagnostic criteria for sarcopenic obesity were adopted based on pre-defined body composition cut points of this condition, as well as individual definitions of low muscle mass (per previous sentence) and obesity according to a previous systematic literature search (Johnson Stoklossa et al., 2017). Although differences in body composition among ethnicities may exist, definitions were not excluded based on ethnicity to ensure our findings are inclusive and can inform future research regarding performance of each published definition, a similar approach to others (Batsis et al., 2013; Khor et al., 2020). Many previous publications that established sarcopenic obesity cut points were developed from individuals with a wide variety of ethnicities (Baumgartner et al., 1998, 2004; Newman et al., 2003; Levine and Crimmins, 2012; Prado et al., 2014; Batsis et al., 2015; Siervo et al., 2015).

Eleven studies that defined sarcopenic obesity based upon body composition derived from DXA were used in the present analysis (Baumgartner et al., 1998, 2004; Newman et al., 2003; Zoico et al., 2004; Bouchard et al., 2009; Kim et al., 2009; Levine and Crimmins, 2012; Prado et al., 2014; Batsis et al., 2015; Oh et al., 2015; Siervo et al., 2015). As many of these publications developed multiple definitions of sarcopenic obesity, a total of 29 definitions were applied to the CLSA sample.

Each definition of low muscle mass and obesity used sex-specific cut-points, except for those that defined obesity using BMI. Linear regression with ASM, height, FM, with (Kim et al., 2009) or without (Newman et al., 2003) age were used to determine low muscle mass prevalence using residuals. In this method, cutpoints were determined based on relative distance from the regression line; positive residuals indicate relatively higher ASM while lower residuals indicate relatively lower ASM.

Two publications (Zoico et al., 2004; Kim et al., 2009) calculated skeletal muscle percent as:

$$\frac{\left(1.13\,x\,\mathrm{ASM}\right)-\left(0.02\,x\,\mathrm{age}\right)+\left(0.61\,x\,\mathrm{sex}\right)+0.97}{\mathrm{Body}\,\mathrm{weight}}\times 100$$

where ASM and body weight are in kilograms, age is in years and sex = 0 for female and 1 for male (Kim et al., 2002).

Abnormal body composition was also identified using centiles of FM:FFM and TrFM:ASM proposed by Siervo et al. (2015). Body composition phenotypes as proposed by Prado et al. (2014) (low adiposity/high muscularity, high adiposity/high muscularity, low adiposity/low muscularity, high adiposity/low muscularity) using deciles of ASMI and FM index were also explored. The prevalence of abnormal body composition phenotypes using the Prado et al. cut-points were described in more depth and across age groups because these definitions are specific for sex, age and BMI and were developed using a large sample of North Americans, who presumably have similar characteristics to individuals in the CLSA dataset.

### Strength and Physical Performance

Handgrip strength was measured using a digital grip dynamometer (Tracker Freedom JTECH Medical; Midvale, Utah, United States). Participants sat in a straight-backed chair with feet on the floor and upper arms close to the body with the elbow of the dominant hand at 90 degrees flexion. After a practice maximal squeeze of the dynamometer, participants completed three maximal squeezes with 15 s between each trial. The highest measure from the dominant hand was used in this analysis. Low handgrip strength was defined as < 16 kg in females and < 27 kg in males, in line with the updated recommendations proposed by the European Working Group on Sarcopenia in Older People (Cruz-Jentoft et al., 2019).

Participants completed a 4-meter gait speed test to assess physical performance. A practice trial was completed before each test. Participants were instructed to walk as fast as possible through a straight 4-meter path marked on the floor and asked to stop a few steps after the finish line. The time taken to complete the course was recorded using a stopwatch. Speed was calculated as the 4-meter length of the path divided by the time to complete the test (m/s). Slow gait speed was defined as < 0.8 m/s (Cruz-Jentoft et al., 2019).

### Statistical Analysis

Descriptive statistics were conducted to characterize subject demographics, anthropometrics, and body composition. For continuous variables such as age and BMI, mean ± standard deviation (SD) were reported and normality was assessed using the Anderson-Darling Normality test. For variables with discrete quantities, such as sex and binary outcomes (yes/no) for each body composition definition, frequencies and proportions were reported. No sampling or analytical weight were applied to our data. Differences in continuous variables between males and females were assessed using independent samples *t*-test. Pearson correlation was used to describe the relationship between body weight with muscle mass. Chi-square tests were used to describe the relationship between each definition of sarcopenic obesity with low handgrip strength (categorized into binary variables based on sex-specific cut-points) and slow gait speed. All analyses reported 2-sided *p*-values with significance levels of 0.05. STATA^®^14 (StataCorp LLC, College Station, TX) was used for data analysis.

## Results

### Subject Characteristics

A total of 11,803 adults ≥ 65 years old were included in the present analysis (*n* = 5,856, 49.6% males; *n* = 5,947, 50.4% females). Of those, 2,532 (21.5%) had Type II diabetes mellitus, 5,798 (49.1%) had hypertension, 2,269 (19.2%) had any type of heart disease, and 521 (4.4%) were current smokers. Most (*n* = 11,745, 99.5%) had measures of BMI, 11,196 (94.9%) had measures of body composition, 10,669 (90.4%) had measures of handgrip strength, and 11,655 (98.7%) had gait speed. Two individuals’ body composition data was excluded due to unfeasible values (i.e., LST greater than body weight). The average age of the entire sample was 73.1 ± 5.7 years and most individuals had a BMI in the World Health Organization-defined overweight (*n* = 5,057, 43.1%) or obese (*n* = 3,342, 28.5%) categories with a small proportion in considered underweight (*n* = 96, 0.1%). Most individuals reported being married or in a common law partnership (*n* = 7,354, 62.3%) and 21.1% (*n* = 2,489) reported a university degree or certificate above a bachelor’s degree. As expected, differences in height, weight, and body composition were apparent between males and females, Table 1.

**TABLE 1:** Demographic, anthropometric, and body composition characteristics of 11,803 Caucasian Canadians ≥ 65 years old.

Variable	Females	Males	*p*
Age, years	73 ± 6	73 ± 6	0.710
	68 – 78	68 – 78	
Height, cm	159.9 ± 6.3	174.0 ± 6.8	< 0.001
	155.6 – 164.1	169.5 – 178.6	
Weight, kg	71.3 ± 14.9	85.0 ± 14.4	< 0.001
	61.9 – 79.3	75.1 – 92.7	
BMI, kg/m^2^	27.9 ± 5.7	28.0 ± 4.3	0.180
	23.9 – 30.9	25.1 – 30.3	
Fat mass, kg	29.8 ± 9.6	25.6 ± 8.2	< 0.001
	23.1 – 35.1	20.1 – 30.0	
Fat mass%	40.7 ± 5.9	29.6 ± 5.3	< 0.001
	37.0 – 44.7	26.0 – 32.9	
Fat mass index, kg/m^2^	11.7 ± 3.8	8.5 ± 2.6	< 0.001
	9.1 – 13.7	6.7 – 9.8	
Fat mass:fat-free mass	0.52 ± 0.20	0.51 ± 0.20	0.112
	0.37 – 0.66	0.36 – 0.64	
ASM, kg	17.4 ± 3.0	26.0 ± 3.9	< 0.001
	58.7 – 80.7	23.3 – 28.4	
ASMI, kg/m^2^	6.8 ± 1.1	8.6 ± 1.1	< 0.001
	6.1 – 7.4	7.8 – 9.2	
ASM%	24.7 ± 2.9	30.9 ± 3.1	< 0.001
	22.7 – 26.4	28.8 – 32.9	
ASM:BMI	0.6 ± 0.1	0.9 ± 0.1	< 0.001
	0.6 – 0.7	0.9 – 1.0	

*ASM, Appendicular skeletal muscle; ASMI, appendicular skeletal muscle index; BMI, body mass index. Results are reported as mean ± standard deviation and (interquartile range). *p*-value attained from independent-samples *t*-tests.*

**FIGURE 1:**
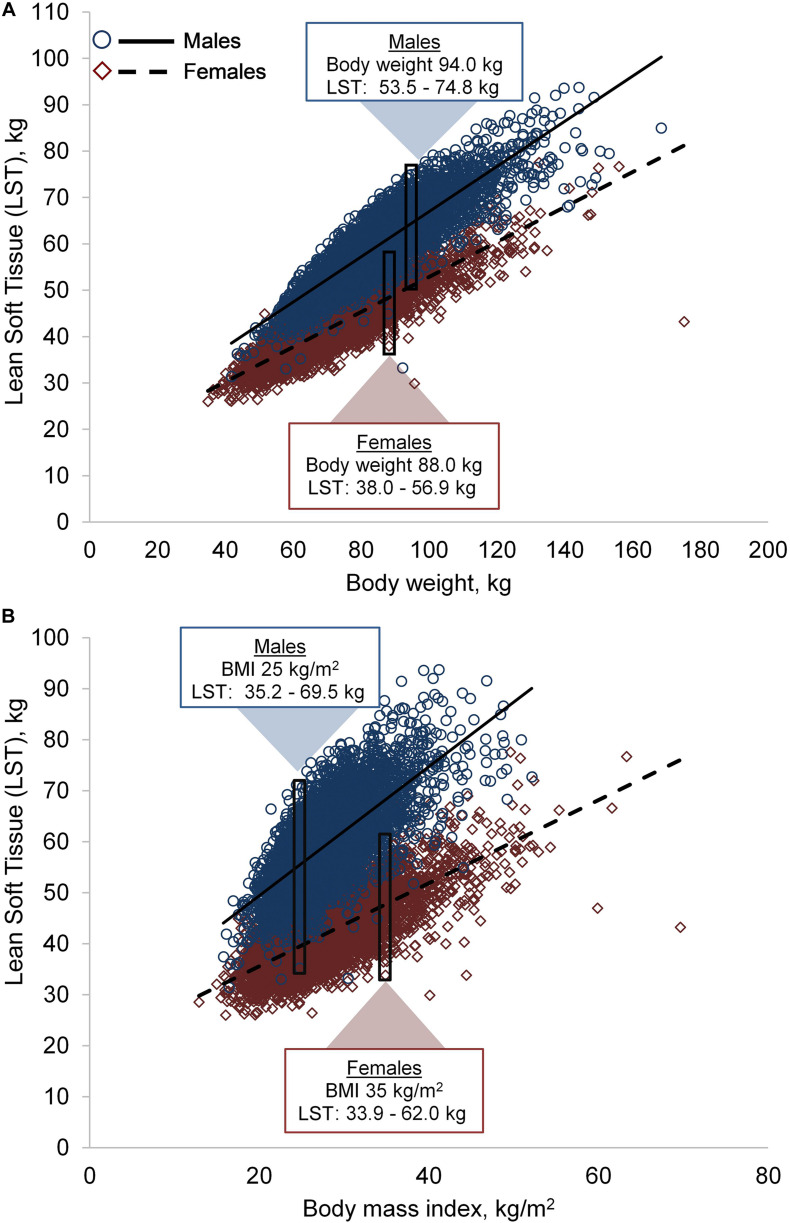
Variability in lean soft tissue (LST) by body weight **(A)** and body mass index (BMI) **(B)**. Males, *n* = 5,585; females, *n* = 5,605. The rectangles and corresponding data are examples of LST variability in two individuals with similar body weight or BMI.

LST was positively correlated to body weight (in both sexes, *r* = 0.88, *p* < 0.001) and BMI (males: *r* = 0.69, *p* < 0.001; females: *r* = 0.72, *p* < 0.001). However, a wide variability in LST was observed across the body weight and BMI spectrum in both sexes, Figures 1A,B. For example, males with a BMI of 25.0 kg/m^2^ had LST ranging between 35.2 and 69.5 kg; likewise, females with a BMI of 35.0 kg/m^2^ had LST ranging from 33.9 to 62.0 kg.

### Prevalence of Sarcopenic Obesity

The prevalence of sarcopenic obesity using each definition are presented in Table 2. Prevalence of sarcopenic obesity ranged from 0.1% based on Newman et al. (2003) to 85.3% based on Kim et al. (2009) in males and 0% based on definitions from Batsis et al. and Zoico et al. (Newman et al., 2003; Zoico et al., 2004; Batsis et al., 2015) to 80.4% based on Kim et al. in females (Kim et al., 2009).

**TABLE 2:** Prevalence of sarcopenic obesity according to different criteria in *n* = 11,803 adults ≥ 65 years old.

	Cut-points				Prevalence (%)	
		
	Males		Females		Males	Females
		
	Low muscle mass	Obesity	Low muscle mass	Obesity		
Batsis et al., 2015	ASM < 19.75	FM% > 25.0	ASM < 15.02	FM% > 35.0	6.5	0.0
	ASM:BMI < 0.789	FM% > 25.0	ASM:BMI < 0.512	FM% > 35.0	10.6	8.2
Baumgartner et al., 1998, 2004	ASMI < 7.26	FM% > 28.0	ASMI < 5.45	FM% > 40.0	5.4	2.6
Bouchard et al., 2009	ASMI < 8.51	FM% ≥ 28.0	ASMI < 6.29	FM% ≥ 35.0	28.7	23.6
Kim et al., 2009	ASMI < 7.40	FM% > 20.2	ASMI < 5.14	FM% > 31.7	11.2	2.0
	ASMI < 8.81	FM% > 20.2	ASMI < 7.36	FM% > 31.7	56.2	64.0
	SM%* < 35.7	FM% > 20.2	SM%* < 30.70	FM% > 31.7	55.0	80.4
	Residuals: −1.87	FM% > 20.2	Residuals: −1.62	FM% > 31.7	85.3	79.3
Levine and Crimmins, 2012	ASM% < 25.72	WC > 102	ASM% < 19.43	WC > 88	4.1	1.5
Newman et al., 2003	ASMI < 7.23	BMI ≥ 30	ASMI < 5.67	BMI ≥ 30	0.1	0.1
	Residuals: −2.29	BMI ≥ 30	Residuals: −1.73	BMI ≥ 30	2.0	4.9
Oh et al., 2015	ASM% < 44.0	BMI ≥ 30	ASM% < 52.0	BMI ≥ 30	25.8	27.9
Prado et al., 2014	ASMI 0-49.99 age, sex, weight adjusted decile				8.0	7.3
	FMI 50-100 age, sex, weight-adjusted decile					
Siervo et al., 2015	FM:FFM ≥ 85th percentile				5.9	46.0
	FM:FFM ≥ 95th percentile				4.9	25.8
	TrFM:ASM ≥ 85th percentile				31.6	10.5
	TrFM:ASM ≥ 95th percentile				23.3	7.2
Zoico et al., 2004	-		SMI < 5.7	BMI > 30	-	0.0
	-		ASMI < 5.45	BMI > 30	-	0.0
	-		ASMI 4.7–5.6	BMI > 30	-	0.1
	-		ASMI < 4.7	BMI > 30	-	0.0
	-		SM%* 23–27	BMI > 30	-	18.2
	-		SM%* < 23	BMI > 30	-	4.7
	-		SMI* < 5.7	BMI > 30	-	0.7
	-		ASMI < 5.45	BMI > 30	-	1.3
	-		ASMI 4.7–5.6	BMI > 30	-	2.0
	-		ASMI < 4.7	BMI > 30	-	0.1
	-		SM%* 23–27	BMI > 30	-	25.8
	-		SM%* < 23	BMI > 30	-	6.2

ASM, appendicular skeletal muscle, in kg; ASM%, percent of body weight as appendicular skeletal muscle; ASMI, appendicular skeletal muscle index, in kg/m^2^; BMI, body mass index, in kg/m^2^; FFM, fat-free mass, in kg; FM, fat mass, in kg; FM%, percent of body weight as fat mass; FMI, fat mass index, in kg/m^2^; SM%, percent of body weight as skeletal muscle; SMI, skeletal muscle index, in kg/m^2^; TrFM, trunk fat mass; WC, waist circumference, in cm. *Skeletal muscle attained from the following equation (Kim et al., 2002): (1.13 × ASM) – (0.02 × age) + (0.61 × sex) + 0.97, where sex is 0 for females and 1 for males.

Supplementary Table 1 includes the prevalence for each individual phenotype (depicting low muscle mass or obesity). In males, prevalence of low muscle mass ranged from 4.6% based on the definition from Levine and Crimmons (Levine and Crimmins, 2012) to 95.1% based on the definition from Oh et al. (2015). In females, low muscle mass prevalence ranged from 0.5% using cut-points from Zoico et al. (2004) to 94.0% using Oh et al. (2015). At least 27.2% of males and 29.7% of females had obesity based on the definition as BMI 30 kg/m^2^ or higher (Newman et al., 2003; Oh et al., 2015; Siervo et al., 2015). The prevalence of obesity was generally higher if using body composition assessments (i.e., FM or % FM) or waist circumference as opposed to BMI-based definitions.

**FIGURE 2:**
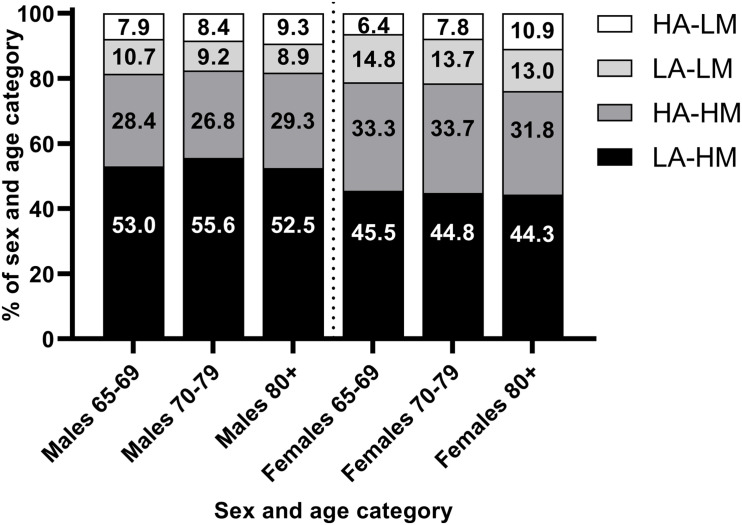
Prevalence of different body composition phenotypes by sex and age group. HA, high adiposity; LM, low muscularity; LA, low adiposity; LM, low muscularity. High and low adiposity and muscularity defined as > or < 50th decile of age-, sex-, weight-adjusted fat mass index (adiposity) and appendicular skeletal muscle index (muscularity) from Prado et al. (2014).

The most prevalent body composition phenotype based on the framework proposed by Prado et al. (2014) was low adiposity and high muscularity (54.2% males, 45.0% females), followed by high adiposity and high muscularity (27.8% males, 33.2 females). High adiposity and low muscularity was present only in a minority of individuals (8.4% males, 7.8% females) and the prevalence of low adiposity and low muscle across all subgroups was higher than the prevalence of underweight (0.1%). However, the prevalence of this body composition phenotype increased across age groups, Figure 2.

### Relationship Between Sarcopenic Obesity and Physical Performance and Strength

As shown in Table 3, there was a significant relationship between low handgrip strength and sarcopenic obesity from most definitions (males: 14/17 definitions [82.4%]; females: 21/29 definitions [72.4%]). In other words, individuals with sarcopenic obesity were more likely to have low handgrip strength than those without sarcopenic obesity. However, few definitions of sarcopenic obesity were related to slow gait speed (males: 1/17 definitions [6.7%]; females: 2/29 [6.9%]) (Newman et al., 2003; Zoico et al., 2004; Batsis et al., 2015).

**TABLE 3:** Relationship of sarcopenic obesity (SO) definitions with handgrip strength and gait speed.

	Cut-points		Low handgrip strength		Slow gait speed	
			
	Males	Females	Males	Females	Males	Females
			*n* = 5,446	*n* = 5,223	*n* = 5,789	*n* = 5,866
			*p*		*p*	
				
Batsis et al., 2015	ASM < 19.75	ASM < 15.02	< 0.001	N/A**	0.627	N/A**
	FM% > 25.0	FM% > 35.0				
	ASM:BMI < 0.789	ASM:BMI < 0.512	< 0.001	<0.001	0.276	0.026
	FM% > 25.0	FM% > 35.0				
Baumgartner et al., 1998, 2004	ASMI < 7.26	ASMI < 5.45	< 0.001	<0.001	0.298	0.911
	FM% > 28.0	FM% > 40.0				
Bouchard et al., 2009	ASMI < 8.51	ASMI < 6.29	< 0.001	<0.001	0.355	0.229
	FM% ≥ 28.0	FM% ≥ 35.0				
Kim et al., 2009	ASMI < 7.40	ASMI < 5.14	< 0.001	<0.001	0.302	0.885
	FM% > 20.2	FM% > 31.7				
	ASMI < 8.81	ASMI < 7.36	< 0.001	<0.001	0.419	0.193
	FM% > 20.2	FM% > 31.7				
	SM%* < 35.7	SM%* < 30.70	< 0.001	<0.001	0.663	0.063
	FM% > 20.21	FM% > 31.7				
	Residuals: −1.87	Residuals: −1.62	< 0.001	<0.001	0.662	0.541
	FM% > 20.2	FM% > 31.7				
Levine and Crimmins, 2012	ASM% < 25.72	ASM% < 19.43	< 0.001	<0.001	0.411	0.552
	WC > 102	WC > 88				
Newman et al., 2003	ASMI < 7.23	ASMI < 5.67	< 0.001	<0.001	0.011	N/A***
	BMI ≥ 30	BMI ≥ 30				
	Residuals: −2.29	Residuals: −1.73	< 0.001	<0.001	0.661	0.213
	BMI ≥ 30	BMI ≥ 30				
Oh et al., 2015	ASM% < 44.0	ASM% < 52.0	0.918	0.247	0.551	0.882
	BMI ≥ 30	BMI ≥ 30				
Prado et al., 2014	ASMI 0–49.99 age, sex, weight-adjusted decile		< 0.001	<0.001	0.854	0.074
	FMI 50–100 age, sex, weight-adjusted decile					
Siervo et al., 2015	FM:FFM ≥ 85th percentile		< 0.001	0.258	0.626	0.670
	FM:FFM ≥ 95th percentile		0.002	0.001	0.762	0.079
	TrFM:ASM ≥ 85th percentile		0.632	0.110	0.332	0.364
	TrFM:ASM ≥ 95th percentile		0.747	0.347	0.692	0.077
Zoico et al., 2004	-	SMI < 5.7	-	0.008	-	N/A***
		BMI > 30				
	-	ASMI < 5.45	-	< 0.001	-	N/A***
		BMI > 30				
	-	ASMI 4.7–5.6	-	< 0.001	-	N/A***
		BMI > 30				
	-	ASMI < 4.7	-	N/A**	-	N/A**
		BMI > 30				
	-	SM% 23–27*	-	0.542	-	0.369
		BMI > 30				
	-	SM% < 23*	-	< 0.001	-	0.193
		BMI > 30				
	-	SMI < 5.7*	-	< 0.001	-	0.285
		BMI > 30				
	-	ASMI < 5.45	-	< 0.001	-	0.296
		BMI > 30				
	-	ASMI 4.7–5.6	-	< 0.001	-	0.166
		BMI > 30				
	-	ASMI < 4.7	-	0.178	-	N/A***
		BMI > 30				
	-	SM% 23–27*	-	0.004	-	0.948
		BMI > 30				
	-	SM% < 23*	-	< 0.001	-	0.030
		BMI > 30				

Low handgrip strength was defined as < 27 kg in males and < 16 kg in females. Slow gait speed was defined as < 0.8 m/s in both sexes. *P*-values attained from Chi-Square. ASM, appendicular skeletal muscle, in kg; ASM%, percent of body weight as appendicular skeletal muscle; ASMI, appendicular skeletal muscle index, in kg/m^2^; BMI, body mass index, in kg/m^2^; FFM, fat-free mass, in kg; FM, fat mass, in kg; FM%, percent of body weight as fat mass; FMI, fat mass index, in kg/m^2^; SM%, percent of body weight as skeletal muscle; SMI, skeletal muscle index, in kg/m^2^; TrFM, trunk fat mass; WC, waist circumference, in cm. *Skeletal muscle attained from the following equation (Kim et al., 2002): (1.13 × ASM) – (0.02 × age) + (0.61 × sex) + 0.97, where sex is 0 for female and 1 for male. **not applicable due to *n* = 0 with sarcopenic obesity. ***not applicable due to *n* = 0 with slow gait speed.

## Discussion

Identifying the most appropriate definition of sarcopenic obesity for a given population may help improve resource allocation, awareness of the condition, and prevention of physical performance decline in aging adults. This investigation compared definitions of sarcopenic obesity in a large cohort of older Canadian adults and explored the relationship of sarcopenic obesity with grip strength and gait speed. Our results revealed substantial heterogeneity among definitions, which resulted in varied prevalence of sarcopenic obesity in the CLSA cohort, ranging from approximately 0% to 85%. With stratification by age, sex, and BMI, sarcopenic obesity increased across age categories. Furthermore, sarcopenic obesity was frequently associated with low handgrip strength, but not slow gait speed.

In our study, sarcopenic obesity prevalence among older adults aged 65+ years in the CLSA cohort was substantially lower than previously reported in a similar group of individuals residing in the United States using a variety of definitions (Batsis et al., 2013). For example, using cut-points proposed by Baumgartner et al., the prevalence of sarcopenic obesity from our study was approximately 3-times lower in males and 5-times lower in females compared to those in the National Health and Nutrition Examination Survey (Batsis et al., 2013). Differences in prevalence may reflect true variation between Canadian and American geriatric populations or may be a consequence of inclusion of all ethnicities in the analysis by Batsis et al. (2013). Regardless, sarcopenic obesity prevalence is contingent upon the selected cut-point because our analysis and others (Batsis et al., 2013; Johnson Stoklossa et al., 2017; Donini et al., 2019) report high variations in the prevalence of this condition across various definitions.

Variation in the prevalence of abnormal body composition phenotypes may be partially explained by differences in reference populations and methods used to develop definitions for each individual condition (i.e., low muscle mass and obesity) and sarcopenic obesity. Aspects such as age and ethnicity affect body composition and the subsequent consensus of the optimal diagnostic definition. For example, the definition put forth by Kim et al. (2009) was developed using a dataset of Asian adults and, within the CLSA cohort, resulted in high rates of obesity and sarcopenic obesity. Generally, muscle mass is highest in African Americans, followed by Caucasians, Hispanics, and Asians, while percent body fat is highest among Asian subjects (Wang et al., 1994; Silva et al., 2010). Therefore, consideration of ethnicity in the selection of body composition definitions is ideal, although this was not possible in the present analysis due to the low number of subjects who self-identified with ethnicities other than Caucasian.

Slight differences in body composition cut-points also contribute to dissimilar prevalence rates of abnormal body composition phenotypes. For example, two comparable definitions of sarcopenic obesity proposed by Zoico et al. (2004) resulted in prevalence ranging from 6.2 to 25.8% using skeletal muscle percent < 23% versus 23–27%, respectively (both with FM% ≥ 42.9%). Such substantial variation from small differences in cut-points highlights the importance of selecting body composition definitions that are appropriate for specific individuals or populations and have reasonable specificity and sensitivity.

As exemplified here and elsewhere (Frankenfield et al., 2001) anthropometric-based measurements are modestly related to body composition. For example, LST (in which muscle mass is a main compartment) may vary up to almost two-times among males and females with the same BMI (Figure 1B). Several definitions of sarcopenic obesity use BMI to define obesity. However, these definitions consistently resulted in lower prevalence rates of obesity compared to those that used DXA-assessed FM. This pattern illustrates the poor sensitivity of anthropometric variables to detect body fat (Romero-Corral et al., 2008) and highlights the limited utility of body weight to characterize body composition. Notably, age-related loss of height and increased propensity toward central adiposity (which is associated with adverse cardiometabolic outcomes and is not captured by BMI) alter the relationship between FM and BMI (Batsis and Zagaria, 2018). A BMI that is considered ‘overweight’ (∼27.5 kg/m^2^) is associated with lower all-cause mortality in older adults (Winter et al., 2014). Therefore, traditional BMI cut points for obesity may be inappropriate in geriatric populations, although there is no consensus on what BMI cut-points are ideal. More accurate tools that are accessible in clinical settings are needed to facilitate early identification of abnormal body composition and develop strategies to mitigate muscle loss and FM gain in older individuals.

Using cut-points adjusted for age, sex, and BMI (Prado et al., 2014), the most common body composition phenotype was high muscularity-low adiposity. Notably, however, over 15% of both males and females had low muscularity across age groups. The prevalence of low muscularity-high adiposity was also higher among older males and females. Therefore, although fewer individuals had low muscle mass, a moderate proportion of older Canadians may be at risk for adverse outcomes associated with low muscle mass, which likely increases with senescence.

There is currently no evidence or consensus that physical function or performance outcomes should be incorporated into sarcopenic obesity definitions (Donini et al., 2019). We therefore examined handgrip strength and walking speed in relation to sarcopenic obesity as a hypothesis-generating, exploratory analysis. Interestingly, handgrip strength was frequently related to sarcopenic obesity, while this was not the case with walking speed. Low muscle mass is associated with increased risk of physical performance limitations in older adults, but this relationship may be dependent on low muscular strength (Visser et al., 2005). High adiposity may also be a stronger predictor of physical function limitations in older adults. For example, in the Nutrition as a Determinant of Successful Aging study, obesity (defined as ≥ 35% and ≥ 28% FM in females and males, respectively) was more strongly related to lower physical performance than low muscle mass among 904 community dwelling older adults (Bouchard et al., 2009). In other words, poor physical performance may only arise after the development of low muscle mass, reduced strength, or higher adiposity. Notably we used the generic term “muscle mass” to depict different compartments and its derivatives as mentioned earlier in this paper. Given the importance of handgrip strength in predicting deleterious health outcomes (Leong et al., 2015) and the ease of use in clinical settings, it may be used as a risk stratification tool to identify individuals with potentially low muscle mass (Cruz-Jentoft et al., 2019). Considering adults in the CLSA cohort were community dwelling adults, it is likely that body composition and strength in this cohort have not yet deteriorated to the point of negatively impacting gait speed [i.e., having ‘severe sarcopenia’(Cruz-Jentoft et al., 2019)]. Future investigations that are specifically designed to generate sarcopenic obesity definitions may consider using hand grip strength.

Although this is currently the largest investigation of sarcopenic obesity prevalence in older Canadian adults, some limitations should be considered when interpreting the presented findings. The CLSA cohort did not attain direct measurements of ethnicity; rather, we assumed ethnicity based on self-reported cultural background. In this study, we also only included individuals who self-reported as cultural backgrounds likely to be Caucasian ethnicity. However, body composition varies across ethnicities (Wang et al., 1994; Silva et al., 2010); as such, developing ethnicity-specific definitions of abnormal body composition is warranted. Studies that measured body composition using DXA were included due to the availability in the CLSA database and pervasive use of this technique within the literature. However, assessment of muscle mass as measured by novel methods (i.e., D_3_ creatine dilution) may more strongly relate to physical function parameters and should be investigated in the context of sarcopenic obesity in future investigations (Cawthon et al., 2018; Donini et al., 2019; Orwoll et al., 2020). In addition, walking acceleration was not accounted for in the 4-meter gait speed test, which may have increased the time taken to complete the assessment.

## Conclusion

In conclusion, the identification of sarcopenic obesity (and its components, low muscle mass and obesity) is largely dependent on the selected definition among community-dwelling older Canadian adults. Furthermore, sarcopenic obesity often co-occurred with low grip strength but not slow gait speed in this population. These results highlight the importance of selecting an appropriate definition of abnormal body composition phenotypes and reinforces the need to develop population-specific criteria for sarcopenic obesity in older adults.

## Data Availability Statement

The datasets presented in this article are not readily available because the CLSA cannot share data to anyone or anywhere that has not been approved for use, to uphold the privacy and confidentiality of CLSA participants. However, data are available from the Canadian Longitudinal Study on Aging (www.clsa-elcv.ca) for researchers who meet the criteria for access to de-identified CLSA data. Requests to access the datasets should be directed to access@clsa-elcv.ca.

## Ethics Statement

The studies involving human participants were reviewed and approved by Research Ethics Office at the University of Alberta. The patients/participants in the CLSA provided their written informed consent to participate in this study.

## Author Contributions

ID, MS, and CP contributed to conception and design of the study. CP acquired the data. SP and MY performed the analyses. SP wrote the first draft of the manuscript. All authors contributed to manuscript revision, read, and approved the submitted version.

## Conflict of Interest

The authors declare that the research was conducted in the absence of any commercial or financial relationships that could be construed as a potential conflict of interest.

## Supplementary Material

Supplementary Table 1 Prevalence of low muscle mass and obesity in the Canadian Longitudinal Study on Aging (*n* = 11,803 older adults ≥ 65 years old) according to different criteria.

## References

[B1] Bales C. W. Ritchie C. S. (2002). Sarcopenia, weight loss, and nutritional frailty in the elderly. *Annu. Rev. Nutr.* 22 309–323. 10.1146/annurev.nutr.22.010402.102715 12055348

[B2] Batsis J. A. Barre L. K. Mackenzie T. A. Pratt S. I. Lopez-Jimenez F. Bartels S. J. (2013). Variation in the prevalence of sarcopenia and sarcopenic obesity in older adults associated with different research definitions: dual-energy X-ray absorptiometry data from the National Health and Nutrition Examination Survey 1999-2004. *J. Am. Geriatr. Soc.* 61 974–980. 10.1111/jgs.12260 23647372

[B3] Batsis J. A. Mackenzie T. A. Lopez-Jimenez F. Bartels S. J. (2015). Sarcopenia, sarcopenic obesity, and functional impairments in older adults: National Health and Nutrition Examination Surveys 1999-2004. *Nutr. Res.* 35 1031–1039. 10.1016/j.nutres.2015.09.003 26472145 PMC4825802

[B4] Batsis J. A. Zagaria A. B. (2018). Addressing Obesity in Aging Patients. *Med. Clin. North Am.* 102 65–85. 10.1016/j.mcna.2017.08.007 29156188 PMC5724972

[B5] Baumgartner R. N. Koehler K. M. Gallagher D. Romero L. Heymsfield S. B. Ross R. R. (1998). Epidemiology of sarcopenia among the elderly in New Mexico. *Am. J. Epidemiol.* 147 755–763. 9554417 10.1093/oxfordjournals.aje.a009520

[B6] Baumgartner R. N. Wayne S. J. Waters D. L. Janssen I. Gallagher D. Morley J. E. (2004). Sarcopenic obesity predicts instrumental activities of daily living disability in the elderly. *Obes. Res.* 12 1995–2004. 10.1038/oby.2004.250 15687401

[B7] Bouchard D. R. Dionne I. J. Brochu M. (2009). Sarcopenic/obesity and physical capacity in older men and women: data from the Nutrition as a Determinant of Successful Aging (NuAge)-the Quebec longitudinal Study. *Obesity* 17 2082–2088. 10.1038/oby.2009.109 19373219

[B8] Cawthon P. M. Orwoll E. S. Peters K. E. Ensrud K. E. Cauley J. A. Kado D. M. (2018). Strong Relation Between Muscle Mass Determined by D3-creatine Dilution, Physical Performance, and Incidence of Falls and Mobility Limitations in a Prospective Cohort of Older Men. *J. Gerontol. A Biol. Sci. Med. Sci.* 74 844–852. 10.1093/gerona/gly129 29897420 PMC6521914

[B9] Cruz-Jentoft A. J. Bahat G. Bauer J. Boirie Y. Bruyère O. Cederholm T. (2019). Sarcopenia: revised European consensus on definition and diagnosis. *Age Ageing* 48 16–31. 10.1093/ageing/afy169 30312372 PMC6322506

[B10] Donini L. M. Busetto L. Bauer J. M. Bischoff S. Boirie Y. Cederholm T. (2019). Critical appraisal of definitions and diagnostic criteria for sarcopenic obesity based on a systematic review. *Clin. Nutr.* 39 2368–2388. 10.1016/j.clnu.2019.11.024 31813698

[B11] Frankenfield D. C. Rowe W. A. Cooney R. N. Smith J. S. Becker D. (2001). Limits of body mass index to detect obesity and predict body composition. *Nutrition* 17 26–30. 10.1016/s0899-9007(00)00471-8 11165884

[B12] Heymsfield S. B. Peterson C. M. Thomas D. M. Heo M. Schuna J. M. Jr. (2016). Why are there race/ethnic differences in adult body mass index-adiposity relationships? A quantitative critical review. *Obes. Rev.* 17 262–275. 10.1111/obr.12358 26663309 PMC4968570

[B13] Heymsfield S. B. Smith R. Aulet M. Bensen B. Lichtman S. Wang J. (1990). Appendicular skeletal muscle mass: measurement by dual-photon absorptiometry. *Am. J. Clin. Nutr.* 52, 214–218. 10.1093/ajcn/52.2.214 2375286

[B14] Janssen I. Shepard D. S. Katzmarzyk P. T. Roubenoff R. (2004). The healthcare costs of sarcopenia in the United States. *J. Am. Geriatr. Soc.* 52 80–85. 14687319 10.1111/j.1532-5415.2004.52014.x

[B15] Johnson Stoklossa C. A. Sharma A. M. Forhan M. Siervo M. Padwal R. S. Prado C. M. (2017). Prevalence of Sarcopenic Obesity in Adults with Class II/III Obesity Using Different Diagnostic Criteria. *J. Nutr. Metab.* 2017:7307618. 10.1155/2017/7307618 28421144 PMC5380855

[B16] KhorE. Q.LimJ. P.TayL.YeoA.YewS.DingY. Y. (2020). Obesity Definitions in Sarcopenic Obesity: Differences in Prevalence, Agreement and Association with Muscle Function. *J. Frailty Aging* 9 37–43. 10.14283/jfa.2019.28 32150212

[B17] Kim J. Wang Z. Heymsfield S. B. Baumgartner R. N. Gallagher D. (2002). Total-body skeletal muscle mass: estimation by a new dual-energy X-ray absorptiometry method. *Am. J. Clin. Nutr.* 76 378–383. 10.1093/ajcn/76.2.378 12145010

[B18] Kim T. N. Yang S. J. Yoo H. J. Lim K. I. Kang H. J. Song W. (2009). Prevalence of sarcopenia and sarcopenic obesity in Korean adults: the Korean sarcopenic obesity study. *Int. J. Obes.* 33 885–892. 10.1038/ijo.2009.130 19564878

[B19] Kirkland S. A. Griffith L. E. Menec V. Wister A. Payette H. Wolfson C. (2015). Mining a Unique Canadian Resource: The Canadian Longitudinal Study on Aging. *Can. J. Aging* 34 366–377. 10.1017/S071498081500029X 26300192

[B20] Lear S. A. Kohli S. Bondy G. P. Tchernof A. Sniderman A. D. (2009). Ethnic Variation in Fat and Lean Body Mass and the Association with Insulin Resistance. *J. Clin. Endocrinol. Metab.* 94 4696–4702. 10.1210/jc.2009-1030 19820012

[B21] Leong D. P. Teo K. K. Rangarajan S. Lopez-Jaramillo P. Avezum A. J. Orlandini A. (2015). Prognostic value of grip strength: findings from the Prospective Urban Rural Epidemiology (PURE) study. *Lancet* 386 266–273. 10.1016/S0140-6736(14)62000-6 25982160

[B22] Levine M. E. Crimmins E. M. (2012). The impact of insulin resistance and inflammation on the association between sarcopenic obesity and physical functioning. *Obesity* 20 2101–2106. 10.1038/oby.2012.20 22310233 PMC3527629

[B23] Newman A. B. Kupelian V. Visser M. Simonsick E. Goodpaster B. Nevitt M. (2003). Sarcopenia: alternative definitions and associations with lower extremity function. *J. Am. Geriatr. Soc.* 51 1602–1609. 10.1046/j.1532-5415.2003.51534.x 14687390

[B24] Oh C. Jho S. No J.-K. Kim H.-S. (2015). Body composition changes were related to nutrient intakes in elderly men but elderly women had a higher prevalence of sarcopenic obesity in a population of Korean adults. *Nutr. Res.* 35 1–6. 10.1016/j.nutres.2014.07.018 25524331

[B25] Orwoll E. S. Peters K. E. Hellerstein M. Cummings S. R. Evans W. J. Cawthon P. M. (2020). The Importance of Muscle Versus Fat Mass in Sarcopenic Obesity: A Re-evaluation Using D3-Creatine Muscle Mass Versus DXA Lean Mass Measurements. *J. Gerontol. A Biol. Sci. Med. Sci.* 75 1362–1368. 10.1093/gerona/glaa064 32436565 PMC7302180

[B26] Prado C. M. M. Siervo M. Mire E. Heymsfield S. B. Stephan B. C. M. Broyles S. (2014). A population-based approach to define body-composition phenotypes. *Am. J. Clin. Nutr.* 99 1369–1377. 10.3945/ajcn.113.078576 24760978

[B27] Prado C. M. M. Wells J. C. K. Smith S. R. Stephan B. C. M. Siervo M. (2012). Sarcopenic obesity: A Critical appraisal of the current evidence. *Clin. Nutr.* 31 583–601. 10.1016/j.clnu.2012.06.010 22809635

[B28] Raina P. S. Wolfson C. Kirkland S. A. Griffith L. E. Oremus M. Patterson C. (2009). The Canadian longitudinal study on aging (CLSA). *Can. J.* 28 221–229. 10.1017/S0714980809990055 19860977

[B29] Romero-Corral A. Somers V. K. Sierra-Johnson J. Thomas R. J. Bailey K. R. Collazo-Clavell M. L. (2008). Accuracy of Body Mass Index to Diagnose Obesity In the US Adult Population. *Int. J. Obes.* 32 959–966. 10.1038/ijo.2008.11 18283284 PMC2877506

[B30] Roubenoff R. (2004). Sarcopenic obesity: the confluence of two epidemics. *Obes. Res.* 12 887–888. 10.1038/oby.2004.107 15229325

[B31] Schrager M. A. Metter E. J. Simonsick E. Ble A. Bandinelli S. Lauretani F. (2007). Sarcopenic obesity and inflammation in the InCHIANTI study. *J. Appl. Physiol.* 102 919–925. 10.1152/japplphysiol.00627.2006 17095641 PMC2645665

[B32] Siervo M. Prado C. M. Mire E. Broyles S. Wells J. C. K. Heymsfield S. (2015). Body composition indices of a load-capacity model: gender- and BMI-specific reference curves. *Public Health Nutr.* 18 1245–1254. 10.1017/S1368980014001918 25221994 PMC10272998

[B33] Silva A. M. Shen W. Heo M. Gallagher D. Wang Z. Sardinha L. B. (2010). Ethnicity-related skeletal muscle differences across the lifespan. *Am. J. Human Biol.* 22 76–82. 10.1002/ajhb.20956 19533617 PMC2795070

[B34] Statistics Canada (2019a). *Canada’s Population Estimates: Age and Sex, July 1, 2018*. Available online at: https://www150.statcan.gc.ca/n1/daily-quotidien/190125/dq190125a-eng.htm (accessed June 17, 2019).

[B35] Statistics Canada (2019b). *Table 13-10-0096-20 Body Mass Index, Overweight or Obese, Self-Reported, Adult, Age Groups (18 Years and Older). Canadian Community Health Survey - Annual Component*. Available online at: https:// www150.statcan.gc.ca/t1/tbl1/en/tv.action?pid=1310009620 (accessed June 17, 2019).

[B36] Visser M. Goodpaster B. H. Kritchevsky S. B. Newman A. B. Nevitt M. Rubin S. M. (2005). Muscle mass, muscle strength, and muscle fat infiltration as predictors of incident mobility limitations in well-functioning older persons. *J. Gerontol. A Biol. Sci. Med. Sci.* 60 324–333. 10.1093/gerona/60.3.324 15860469

[B37] Visser M. Schaap L. A. (2011). Consequences of sarcopenia. *Clin. Geriatr. Med.* 27 387–399. 10.1016/j.cger.2011.03.006 21824554

[B38] Wang J. Thornton J. C. Russell M. Burastero S. Heymsfield S. Pierson R. N. J. (1994). Asians have lower body mass index (BMI) but higher percent body fat than do whites: comparisons of anthropometric measurements. *Am. J. Clin. Nutr.* 60 23–28. 10.1093/ajcn/60.1.23 8017333

[B39] Winter J. E. MacInnis R. J. Wattanapenpaiboon N. Nowson C. A. (2014). BMI and all-cause mortality in older adults: a meta-analysis. *Am. J. Clin. Nutr.* 99 875–890. 10.3945/ajcn.113.068122 24452240

[B40] World Health Organization (2015). *WHO | Deaths from NCDs*. Geneva: WHO.

[B41] Zhang X. Xie X. Dou Q. Liu C. Zhang W. Yang Y. (2019). Association of sarcopenic obesity with the risk of all-cause mortality among adults over a broad range of different settings: a updated meta-analysis. *BMC Geriatr.* 19:183. 10.1186/s12877-019-1195-y 31269909 PMC6610788

[B42] Zoico E. Di Francesco V. Guralnik J. M. Mazzali G. Bortolani A. Guariento S. (2004). Physical disability and muscular strength in relation to obesity and different body composition indexes in a sample of healthy elderly women. *Int. J. Obes. Relat. Metab. Disord.* 28 234–241. 10.1038/sj.ijo.0802552 14708033

