# Supplementary material for: Prevalence of Sarcopenic Obesity Using Different Definitions and the Relationship With Strength and Physical Performance in the Canadian Longitudinal Study of Aging
Source: Front Physiol. 2021 Jan 21;11:583825. doi: 10.3389/fphys.2020.583825 (PMC7859259; doi:10.3389/fphys.2020.583825)
Supplement: Supplementary Table 1 — Prevalence of low muscle mass and obesity in the Canadian Longitudinal Study on Aging (n = 11,803 older adults ≥ 65 years old) according to different criteria. [file Table_1.docx]

Purcell et al. Prevalence of Sarcopenic Obesity using Different Definitions and the Relationship with Strength and Physical Performance in the Canadian Longitudinal Study of Aging

**Supplementary Table 1:** Prevalence of low muscle mass and obesity in the Canadian Longitudinal Study on Aging (n=11,803 older adults ≥65 years old) according to different criteria.

| **Reference** | **Low muscle mass** | | | | **Obesity** | | | |
| --- | --- | --- | --- | --- | --- | --- | --- | --- |
|  | **Definition** | | **Prevalence (%)** | | **Definition** | | **Prevalence (%)** | |
|  | **Males** | **Females** | **Males** | **Females** | **Males** | **Females** | **Males** | **Females** |
| Batsis et al. 2015 | ASM <19.75 | ASM <15.02 | 22.0 | 3.2 | FM% > 25.0 | FM% >35.0 | 80.7 | 83.3 |
|  | ASM:BMI <0.789 | ASM:BMI <0.512 | 10.6 | 8.2 | FM% > 25.0 | FM% >35.0 | 80.7 | 83.3 |
| Baumgartner et al., 1998, 2004 | ASMI <7.26 | ASMI <5.45 | 9.3 | 6.6 | FM% >28.0 | FM% >40.0 | 61.0 | 57.7 |
| Bouchard et al. 2009 | ASMI <8.51 | ASMI <6.29 | 48.7 | 31.7 | FM% ≥28.0 | FM% ≥35.0 | 61.0 | 83.3 |
| Kim et al. 2009 | ASMI <7.40 | ASMI <5.14 | 12.0 | 2.7 | FM% >20.2 | FM% >31.7 | 96.8 | 92.6 |
|  | ASMI <8.81 | ASMI <7.36 | 58.4 | 70.4 | FM% >20.2 | FM% >31.7 | 96.8 | 92.6 |
|  | SM% <35.71* | SM% <30.70* | 55.7 | 81.4 | FM% >20.2 | FM% >31.7 | 96.8 | 92.6 |
|  | Residuals: -1.87 | Residuals: -1.62 | 87.6 | 84.2 | FM% >20.2 | FM% >31.7 | 96.8 | 92.6 |
| Levine, Crimmons 2012 | ASM% <25.72 | ASM% <19.43 | 4.6 | 1.6 | WC >102 | WC >88 | 42.9 | 51.1 |
| Newman et al. 2003 | ASMI <7.23 | ASMI <5.67 | 8.6 | 11.6 | BMI ≥30 | BMI ≥30 | 27.2 | 29.7 |
|  | Residuals: -2.29 | Residuals: -1.73 | 10.9 | 15.6 | BMI ≥30 | BMI ≥30 | 27.2 | 29.7 |
| Oh et al. 2015 | ASM% <44.0 | ASM% <52.0 | 95.1 | 94.0 | BMI ≥30 | BMI ≥30 | 27.2 | 29.7 |
| Prado et al. 2014 | ASMI 0-49.99 (age, sex, BMI decile) | | 17.1 | 20.5 | FMI 50-100 (age, sex, BMI decile) | | 34.3 | 38.5 |
| Zoico et al. 2004 | - | SMI <5.7 | - | 3.6 | - | BMI >30 | - | 29.7 |
|  | - | ASMI <5.45 | - | 6.6 | - | BMI >30 | - | 29.7 |
|  | - | ASMI 4.7-5.6 | - | 9.3 | - | BMI >30 | - | 29.7 |
|  | - | ASMI <4.7 | - | 0.5 | - | BMI >30 | - | 29.7 |
|  | - | SM% 23-27* | - | 42.5 | - | BMI >30 | - | 29.7 |
|  | - | SM% <23* | - | 6.6 | - | BMI >30 | - | 29.7 |
|  | - | SMI <5.7* | - | 3.6 | - | FM% ≥42.9 | - | 37.2 |
|  | - | ASMI <5.45 | - | 6.6 | - | FM% ≥42.9 | - | 37.2 |
|  | - | ASMI 4.7-5.6 | - | 9.3 | - | FM% ≥42.9 | - | 37.2 |
|  | - | ASMI <4.7 | - | 0.5 | - | FM% ≥42.9 | - | 37.2 |
|  | - | SM% 23-27* | - | 42.5 | - | FM% ≥42.9 | - | 37.2 |
|  | - | SM% <23* | - | 6.6 | - | FM% ≥42.9 | - | 37.2 |

Note: Siervo *et al*. definitions are not included because low muscle mass and obesity cannot be separated from FM:FFM or trunk fat mass:ASM cut-points. ASM: appendicular skeletal muscle, in kg; ASM%: percent of body weight as appendicular skeletal muscle; ASMI: appendicular skeletal muscle index, in kg/m^2^; BMI: body mass index, in kg/m^2^; FFM: fat-free mass, in kg; FM: fat mass, in kg; FM%, percent of body weight as fat mass; FMI: fat mass index, in kg/m^2^; SM%: percent of body weight as skeletal muscle; SMI: skeletal muscle index, in kg/m^2^; WC: waist circumference, in cm.

*Skeletal muscle attained from the following equation (Kim et al. Am J Clin Nutr 2002; 76:378-383):

(1.13 x ASM) - (0.02 x age) + (0.61 x sex) + 0.97, where sex is 0 for female and 1 for male.
